# ECMO in major burn patients: feasibility and considerations when multiple modes of mechanical ventilation fail

**DOI:** 10.1186/s41038-017-0085-9

**Published:** 2017-06-20

**Authors:** Jason D. Kennedy, Wesley Thayer, Reuben Beuno, Kelly Kohorst, Avinash B. Kumar

**Affiliations:** 1 Department of Anesthesiology and Critical Care, Vanderbilt University Medical Center, 1211 21st Avenue S; Suite 526, Nashville, TN 37212 USA; 2 Department of Plastic Surgery, Vanderbilt University, Nashville, TN USA

**Keywords:** ARDS, Burns, Extra corporeal membrane oxygenation, Rescue ventilation, Conventional ventilation failure, Veno-venous ECMO

## Abstract

**Background:**

We report two cases of acute respiratory distress syndrome in burn patients who were successfully managed with good outcomes with extra corporeal membrane oxygenation (ECMO) after failing multiple conventional modes of ventilation, and review the relevant literature.

**Case presentation:**

The two patients were a 39-year-old male and 53-year-old male with modified Baux Scores of 79 and 78, respectively, with no known inhalation injury. After the initial modified Parkland-based fluid resuscitation and partial escharotomy, both patients developed worsening hypoxemia and acute respiratory distress syndrome.

The hypoxemia continued to worsen on multiple modes of ventilation including volume control, pressure regulated volume control, pressure control, airway pressure release ventilation and volumetric diffusive ventilation. In both cases, the PaO_2_ ≤ 50 mm Hg on a FiO_2_ 100% during the trial of mechanical ventilation. The deterioration was rapid (<12 h since onset of worsening oxygenation) in both cases.

A decision was made to trial the patients on ECMO. Veno-Venous ECMO (V-V ECMO) was successfully initiated following cannulation-under transesophgeal echo guidance—with the dual lumen Avalon® (Maquet, NJ, USA) cannula. ECMO support was maintained for 4 and 24 days, respectively. Both patients were successfully weaned off ECMO and were discharged to rehabilitation following their complex hospital course.

**Conclusion:**

Early ECMO for isolated respiratory failure in the setting on maintained hemodynamics resulted in a positive outcome in our two burn patients suffered from acute respiratory distress syndrome.

## Background

Major burn injury (defined as involving more than 20% total body surface area (TBSA)) is frequently complicated by acute respiratory failure—specifically oxygenation failure and less commonly by failure to ventilate. The incidence of true acute respiratory distress syndrome (ARDS) in the major burn population is reported to be as high as 40% in patients on mechanical ventilation—with about 33% of all major burn patients requiring mechanical ventilation [1]. Inhalation burn injury increases the risk of acute lung injury and prolongs the duration of mechanical ventilation [2]. The options for managing severe ARDS in the major population beyond lung protective ventilation, trial of prone positioning, and early neuromuscular relaxation to aid mechanical ventilation are not well studied. In reality, the viable options narrow quickly in patients who continue to deteriorate on advanced modes of ventilation such as airway pressure release ventilation (APRV) and high-frequency oscillatory ventilation (HFOV).

We report successful rescue of two adult cases of severe ARDS in major burn patients after rapidly failing multiple modes of mechanical ventilation with veno-venous extracorporeal membrane oxygenation (V-V ECMO). Although ECMO in burn patients has been reported in the past, our cases seek to highlight the possible path for expanded use of ECMO in the burn patient population.

## Case presentation

We obtained informed consent from the patients’ families and later from patients when they were deemed able to provide consent to present the individual cases.

### Case 1

A 39-year-old male with no significant past medical history was transferred, intubated and sedated to our burn center, following a 40% TBSA deep partial thickness and full thickness burns to face, head, bilateral upper and lower extremities, perineum, scrotum, and penis. The mechanism of burn was a gasoline fire, following a gasoline container spillage and explosion near a kerosene lamp in a garage.

On admission, the patient underwent an emergent right upper extremity escarotomy with successful return of pulses. Post initial fluid resuscitation, the patient underwent staged grafting of his burns. Following debridement, he underwent xenograft application and partial skin grafting from the trunk to the right arm 32 × 30 cm, right hand size 15 × 15 cm, left hand size 15 × 15 cm, left arm size 20 × 20 cm. He also underwent xenograft application to 30 × 60 cm and to the right leg.

On postoperative day 1, the patient deteriorated into acute respiratory failure. Thick tenacious secretions, prompted an urgent bronchoscopy and bronchoalveolar lavage (BAL). Broad-spectrum antibiotics were initiated to cover for methicillin-resistant Staphylococcus aureus and Gram-negative organism based on the grams stain. The bronchoscopy showed copious secretions in multiple lung fields. The BAL culture had >100,000 CFU streptococcus pneumonia that was resistant to clindamycin. The hypoxemia continued to worsen within hours on volume control (VC) ventilation, pressure regulated volume control (PRVC), pressure control (PC), APRV and on the volumetric diffusive ventilation. A brief summary of the ventilator changes and salient clinical findings are outlined in Table 1. The patient was sedated and neuromuscular blocked once the FiO_2_ requirements escalated >0.8. The acutely worsening and refractory hypoxemia prompted urgent changes to ventilator management. The FiO_2_ requirements escalated from 0.5 immediately post-operation to 1.0 over 4 h. The positive end-expiratory pressure (PEEP) was titrated using constant-flow, pressure-volume curve (PVC) method to prescribe optimal PEEP [3]. The PEEP on conventional modes of ventilation was 15 cm H_2_O before we transitioned to APRV. The peak airway pressures remained >33 cm H_2_O (mean airway pressures 12–16 cm H_2_O). The dynamic compliance was <17 ml/cm H_2_O.

**Table 1:** Summary of patient characteristics and severity of ARDS prior to initiation of ECMO

	Patient 1	Patient 2
*Gender*	Male	Male
*Age*	39	53
*TBSA (%)*	40	25
*Modified Baux Scores*	79	78
*Inhalation injury*	Negative	Negative
*Onset of acute* *respiratory failure*	HOD 3	HOD 17
*Co-morbidities*	Smoker, obesity	Smoker, hypertension,
*Severity of respiratory failure*		
*Lowest* *PaO*_*2*_ *:FiO*_*2*_ *ratio*	49	38
*Maximum attempted* *PEEP*	14	18
*Hypercarbia*	77	109
*Modes of ventilation* *attempted*	SIMV + PC	SIMV + PC
	PRVC	PRVC
	VC	VC
	PC	PC
	APRV	APRV
	VDR	
*Neuromuscular blockade*	Yes	Yes
*Adjunct therapies* *attempted*	Inhaled epoprostenol	Inhaled epoprostenol
*Prone positioning*	Yes	Yes

*APRV* airway pressure release ventilation, *ARDS* acute respiratory distress syndrome, *ECMO* extracorporeal membrane oxygenation, *PC* pressure control, *PEEP* positive end-expiratory pressure, *PRVC* pressure regulated volume control, *SIMV* synchronized intermittent mandatory ventilation, *TBSA* total body surface area*VC* volume control, *VDR* volume diffusive respirator

APRV settings were P high 32, P low 0 and Time high 3.5 s, Time low 0.5 s. Mean airway pressures in APRV were 30 cm H_2_O. The patient continued to deteriorate on APRV with worsening hypercarbia, hypoxemic and acidemic that prompted the change to the volume diffusive respirator (VDR) or volumetric diffusive ventilation. The VDR settings I:E was 3:1, frequency was 585, VDR peak pressure control was 40 cm H_2_O.

Hemodynamically, the patient required four units packed cells along with intermittent vasoactive medications to maintain mean arterial pressure (MAP) > 65 mm Hg during the period of deterioration.

Given his single organ failure, a decision was made to initiate V-V ECMO. The ECMO cannulation was done under transesophageal echocardiographic guidance after a quick evaluation that ruled out significant valular dysfunction and showed a preserved left ventricular ejection fraction. V-V ECMO was initiated and managed for 4 days with no complications. Patient was successfully weaned from ECMO on day 7. The patient was ultimately discharged to a skilled care facility on day 27.

### Case 2

A 53-year-old reportedly healthy African American male was admitted with 25% TBSA partial and full thickness burns to his upper extremities, chest and back following a flash burn from a grease fire. After burn shock resuscitation, he underwent excision/debridement/grafting of his back, upper extremities and chest. His hands and forearms were autografted and upper arms were covered with xenograft.

His post-graft clinical course was complicated by new onset pulmonary infiltrates; fevers and vasopressor dependent septic shock 96 h post admission. His post recovery from vasopressor-dependent shock was complicated by episodes of delirium, early onset pneumonia (Gram-positive cocci) and late onset fevers (day 10–15) of ill-defined etiology (multiple microbiology cultures, fungal cultures were negative but the serum procalcitonin remained positive). His indolent clinical picture and microbiological profile were not aligned. This prompted concern that the source of infection had not cleared with the rounds of antibiotics or we were not adequate covering all pathogens. The infectious disease recommendation was that he completes a course of 14 days of broad-spectrum antibiotics. He was on broad-spectrum antibiotics up to his deterioration.

The mechanical ventilatory course was also rocky for this patient. He was ventilator dependant from day 3 to 14 on a complex trajectory of escalating and deescalating ventilator support. He was weaned to pressure support on day 12 with settings of 20 cm pressure over PEEP and PEEP of 10 cm H_2_O. He continued to be tachypneic with respiratory rates in the upper 20 and 30 ranges. It is worth noting that the patient was extubated for < 24 h prior to his acute deterioration on day 16.

Less than 24 h, post extubation (day 17), the patient deteriorated rapidly requiring urgent re-intubation and high degree of mechanical ventilator support. The patient was neuromuscularly blocked once we transitioned to pressure control ventilation on FiO_2_ 1.0 early in the deterioration episode. Post intubation, he deteriorated rapidly with severely reduced pulmonary dynamic compliance < 8 ml/cm H_2_O (normal > 50 ml/cm H_2_O). Resultant peak pressure were >45 mm Hg.

We cycled through low tidal volume ventilation using VC, PC, PRVC, ARPV, and a trial of prone positioning for twelve hours. This ranged from conventional ventilation (SIMV, PC) to PRVC and APRV. High PEEP prescriptions of 12–16 cm H_2_O, and high FiO_2_ 0.6 -1.0. APRV settings were P high 33, P low 0 and Time high 3.5 s, Time low 0.5 s. Mean airway pressures on APRV were 28 cm H_2_O. The ongoing refractory hypoxemia that was not responding to the ventilator changes precluded longer trials of prone positioning or alternate rescue modes of ventilation like VDR.

He was progressively acidemic from hypercarbia and we could not improve the PaO_2_ > 45 mm Hg on FiO_2_ 1.0 during the prone position trail. He however maintained his hemodynamics and did not require vasopressors at this point in time. Given his isolated respiratory failure we decided to trial him on V-V ECMO. ECMO was successfully initiated and managed for 24 days. The patient was successfully weaned off ECMO and off mechanical ventilation post tracheostomy and was discharged to a nursing facility on hospital day 54.

## Discussion

ARDS continues to have a high mortality worldwide. The pathophysiological hallmark of ARDS is arterial hypoxemia that is refractory to the oxygen therapy, due to pulmonary shunting. In the thermal injury population, the historical incidence of ARDS was reported in the 3–17% range with a significant increase in the incidence to more than 50% if patients require mechanical ventilation. The definition of ARDS further evolved following the recommendations of the 2012 consensus conference—the so-called Berlin definition. In the present classification, ARDS is classified as mild, moderate or severe. The term “acute lung injury” has been removed and the use of PEEP is factored into the diagnosis of ARDS [4]. Mechanical ventilation, pneumonia, and transfusion support have been previously described as being independent risk factors for developing ARDS in the burn patient population [5].

The critical care management of ARDS has also undergone significant changes in the last two decades. In a recent insightful editorial, Vilar et al., outlines that after almost 25 years of research in ARDS, only three strategies have been shown to have beneficial outcomes after randomized controlled trials. These include low tidal volume ventilation, prone positioning and early use of neuromuscular blockade as strategies in the management of ARDS [6]. In the broader armamentarium of the intensivist managing ARDS include lung-protective ventilatory strategies, optimal lung recruitment and application of PEEP (automated pressure-volume loop methods, open lung techniques, transpulmonary pressure methods, etc.), Prone positioning, rescue use of high frequency oscillatory ventilation, use of selective pulmonary vasodilators, use of a conservative fluid management strategy, early and judicious use of neuromuscular blockade and in selective case and ECMO [7].

It is worth noting that there are subsets of ARDS patients who may continue to deteriorate rapidly in spite of multiple options used. The management options in this challenging subset of patients are limited and often without evidence backed by large randomized controlled trials.

In our study, we focus on the role of ECMO and its role in the management of acute refractory hypoxemia and ARDS in the burn patient population. We had exhausted our conventional, second, and third line therapeutic options with our two cases, and were faced with continued deterioration with worsening hypercarbia, acidemia and profound hypoxemia. The isolated respiratory failure (single organ failure) in the setting of preserved hemodynamics guided our decision to initiate ECMO.

ECMO in selected adult patients can be a life saving form of rescue therapy. ECMO has enjoyed resurgence after successful utilizations during the global H1N1 epidemic [8], [9]. ECMO is in essence, cardiopulmonary bypass that has been optimized for weeks rather than hours of operation. It provides biventricular circulatory and pulmonary support for patients experiencing either isolated pulmonary or myocardial failure or both. Conceptually, a typical V-V ECMO circuit has a venous inflow that draws blood from the patient’s venous circulation into a pump, pushes that blood through an oxygenator, and returns the oxygenated blood to the patient’s venous circulation.

The indications for initiating ECMO usually mirror those reported in the CEASAR trail, ie PaO_2_: FiO_2_ < 80, Plateau pressures > 35 mm Hg, FiO_2_ > 90%, pH < 7.2 with uncompensated hypercarbia in a patient with a reversible disease [10, 11]. In the setting of isolated respiratory failure, V-V ECMO helps provide either complete or partial support of the lungs when cardiac output is sufficient.

### Cannulation and considerations when initiating ECMO

Blood flow in the ECMO circuit is driven with centrifugal blood pump driven circuit flow and polymethylpentene low-resistance oxygenators. V-V ECMO was the mode used in both cases. Both patients had vascular cannular inserted via the right internal jugular vein guided by transesophageal echo. The vascular cannula used was the Avalon Elite® cannula (Maquet, NJ, USA). The cannula consisted of two lumens: one lumen allows the deoxygenated blood to drain from the distal and proximal ports, from the inferior vena cava (IVC) and the superior vena cava (SVC), respectively; and a second lumen allows the oxygenated blood to return from the external pump to the right atrium directed toward the tricuspid valve, the right ventricle and diseased lungs. It is paramount to ensure the safe and correct positioning of the cannula. Malpositions and catheter misadventures can be life threatening. This can be done by fluroscopy or guided by transesophageal echo. V-V ECMO can also be initiated with a two cannulae technique (internal jugular and femoral) that is somewhat simpler to initiate but limits patient mobility due to the femoral venous cannulas.

### Critical care management on ECMO

The lack of a venous reservoir in the ECMO circuit necessitates a more active management to optimize the patient’s intravascular volume status. Specific indicators such as relative changes in central venous pressure, the fluctuation of the flow rate (in the centrifugal pumps) over a rather short period of time (second to minutes) may be foretelling of a volume deficit. The use of transthoracic echocardiography is a very useful adjunct to assess for cannula position, fluid volume status and the development of occult right and left ventricular dysfunction while on ECMO. Persistent positive fluid balance is associated with a worse outcome for patients on ECMO; therefore, positive fluid balances are managed aggressively with diuresis or even renal replacement therapy if indicated [12].

These patients frequently will undergo an early tracheostomy on ECMO to facilitate ventilator management and pulmonary toilet. This also helps with patient comfort and allows for sedation management and safe early ambulation in select cases as well. Surgical procedures in patients on EMCO can often be facilitated with the temporary cessation of heparin along with the administration of epsilon aminocaproic acid to help prevent excess bleeding [13].

### Systemic anticoagulation

Most new extracorporeal circuits are heparin bonded. The absence of the reservoir in V-V ECMO permits lower levels of anticoagulation than what is seen with circuits used for cardio-pulmonary bypass in cardiac surgery. Although unfractionated heparin is the most frequently used anticoagulant, there is wide variability in heparin dosing. Frequently aPTT or ACT’s are used to target aPTT of 60– 80 s.

Factor anti Xa levels combined with thromboelastography (TEG) is being more widely adopted in centers to refine anticoagulation management in ECMO patients [14].

### Limitations and logistical considerations of ECMO in burn patients

ECMO is a high resource utilization therapy (personnel, equipment, transfusion support etc.) that may not be widely available across the country [15]. More significantly, there is paucity of evidence regarding efficacy and outcomes in the burn patient population. The period of early burn resuscitation (high volume fluid resuscitation, escharotomies, wound care, coagulopathy) significantly complicates the logistics of ECMO management—generally making early burn patients not ideal candidates. However, for patients who have isolated inhalational injury or isolated respiratory failure, V-V ECMO can be useful to maintain oxygenation and end organ perfusion.

The need for anticoagulation in a patient with large areas of exposed tissue area also complicates the fluid and transfusion needs during early phase of burn management. The presence of significant comorbidities, intracranial pathology, multisystem failure (e.g., burn sepsis), and coagulopathy may be contraindications for initiating ECMO in the burn patient population. The most frequent complication in patients on ECMO is bleeding, with rates ranging from 10 to 30%. Bleeding can occur at the cannula site, into the site of a previous surgery, intrathoracic (including pulmonary hemorrhage), abdominal (including gastrointestinal), or be retroperitoneal in origin. The risk factors of hemorrhage on ECMO include systemic anticoagulation, platelet dysfunction, and dilution of clotting factors [8]. In addition to patient complications, there are also mechanical problems that can occur including oxygenator failure, cannula problems, pump malfunction, or circuit rupture [16]. Our cases highlight the feasibility and potential anticipated challenges of successfully managing ECMO in the burn patient population.

### A brief overview of literature addressing ECMO in burn patients

The number of studies describing the use of ECMO in the burn patient population is limited, as is the level of evidence. A brief list of the publications in the ECMO burn literature are outlined in Table 2. It is worth noting that most of the studies until 2017 were mostly single center retrospective studies and case series. In a recent 2013 systematic review and metaanalysis (one case series and five retrospective studies), the authors commented that the current state of the science of ECMO and burns is based on limited patient numbers and the level of evidence generated was limited [17]. Since the 2013 publication, there have been two newer studies that analyzed the data from national databases of adult and pediatric burn patients who underwent ECMO [18, 19]. The authors of the pediatric study found encouraging survival data in the ECMO-trauma patient population. The results, however, were not specific to burn patients and one has to be cautious about making broad extrapolations based on that particular study. The adult ECMO study published by Nosonov et al., evaluated data from the national burn registry. Their conclusion was that burn-ECMO mortality was comparable to the mortality reported by the Extracorporeal Life Support Organization (ELSO) for non-burn patients. The authors go onto to suggest ECMO as a viable option in selected burn patients [19].

**Table 2:** Examples of studies that have described the use of ECMO in the burn patient population. Studies in italics were part of the systematic review published in 2013

Author	Year	Journal published	*N*	Type of study
Goretsky et al.	*1995*	*J Pediatr Surg*	*5*	*Retrospective*
*O’ Toole et al.*	*1998*	*Burns*	*3*	*Case series*
*Pierre et al.*	*1998*	*J Burn Care Rehabil*	*5*	*Retrospective review*
*Kane et al.*	*1999*	*J Burn Care Rehabil*	*12*	*Retrospective review*
*Masiakos et al.*	*1999*	*Arch Surg*	*2*	*Retrospective review*
*Chou et al.*	*2001*	*Artif Organs*	*3*	*Case series*
*Nehra et al.*	*2009*	*Arch Surg*	*10*	*Retrospective review*
*Askegard et al.*	*2010*	*J Pediatr Surg*	*36*	*Retrospective review*
Asmussen	2013	Burns		Systematic review
Nosanov et al.	2017	J Burn Care Res	30	Retrospective -National Burn Repository
Po-Shun Hsu et al.	2017	Burns	6	Retrospective
^a^Watson JA et al.	2017	J Pediatr Surg	6	Retrospective - National Trauma Databank

^a^Watson et al. described 6 burn patients as part of a larger cohort of ECMO patients

It is challenging to make broad recommendations and criteria for initiating ECMO in burn patients. However, some extrapolations from other ARDS and ECMO studies may offer guidance to patient selection. The largest study of patients on ECMO for acute respiratory failure describes a well-calibrated and discriminatory survival model that is based on the twelve pre-ECMO variables (RESP score; http://www.respscore.com) at the time of ECMO initiation [20]. This model may offer additional data for decision making whether to initiate ECMO in specific burn patients as well. As clinical data accumulates in larger registries such as the ELSO registry, we anticipate burn and thermal injury related ARDS specific data in the future [21]. Developing a selection criteria and standardizing the burn and ECMO management (anticoagulation, volume status, weaning criteria etc.) of these complex patients maybe an area of opportunity in higher volume burn centers. We can anticipate further studies in this domain as the availability and expertise to manage ECMO grows worldwide.

## Conclusions

Refractory hypoxemia can be extremely challenging to manage in the burn patient population. V-V ECMO should be considered in a rapidly deteriorating patient with isolated life threatening refractory hypoxemia and respiratory failure with preserved hemodynamics in the burn patient population.

## Abbreviations

ACT: Activated clotting time
aPTT: Activated partial thromboplastin time
APRV: Airway pressure release ventilation
ARDS: Acute respiratory distress syndrome
ECMO: Extra corporeal membrane oxygenation
ELSO: Extracorporeal Life Support Organization
PEEP: Positive end expiratory pressure
TEG: Thromboelastography
TBSA: Total body surface area
V-V ECMO: Veno-venous extra corporeal membrane oxygenation
